# Drug Resistance Determinants in Clinical Isolates of *Enterococcus faecalis* in Bangladesh: Identification of Oxazolidinone Resistance Gene *optrA* in ST59 and ST902 Lineages

**DOI:** 10.3390/microorganisms8081240

**Published:** 2020-08-14

**Authors:** Sangjukta Roy, Meiji Soe Aung, Shyamal Kumar Paul, Salma Ahmed, Nazia Haque, Emily Rahman Khan, Tridip Kanti Barman, Arup Islam, Sahida Abedin, Chand Sultana, Anindita Paul, Muhammad Akram Hossain, Noriko Urushibara, Mitsuyo Kawaguchiya, Ayako Sumi, Nobumichi Kobayashi

**Affiliations:** 1 Department of Microbiology, Mymensingh Medical College, Mymensingh 2200, Bangladesh; drsangjukta@gmail.com (S.R.); ahmed.salma51@yahoo.com (S.A.); drnaziahaque@gmail.com (N.H.); emily_rahman_khan@yahoo.com (E.R.K.); dr.arup@gmail.com (A.I.); sahidaabedin@gmail.com (S.A.); drchandsultana40@gmail.com (C.S.); panindita37@gmail.com (A.P.); 2 Department of Hygiene, Sapporo Medical University School of Medicine, Sapporo 060-8556, Japan; meijisoeaung@sapmed.ac.jp (M.S.A.); noriko-u@sapmed.ac.jp (N.U.); kawaguchiya@sapmed.ac.jp (M.K.); sumi@sapmed.ac.jp (A.S.); 3 Netrokona Medical College, Netrokona 2400, Bangladesh; drshyamal10@yahoo.com; 4 Medicine Unit, Mymensingh Medical College Hospital, Mymensingh 2200, Bangladesh; drtridipkanti@gmail.com; 5 Imperial Hospital Ltd. Chittagong, Chittagong 4202, Bangladesh; Akram.prof@gmail.com

**Keywords:** *Enterococcus faecalis*, molecular epidemiology, drug resistance, *optrA*, ST

## Abstract

*Enterococcus faecalis* is one of the major causes of urinary tract infection, showing acquired resistance to various classes of antimicrobials. The objective of this study was to determine the prevalence of drug resistance and its genetic determinants for *E. faecalis* clinical isolates in north-central Bangladesh. Among a total of 210 *E. faecalis* isolates, isolated from urine, the resistance rates to erythromycin, levofloxacin, and gentamicin (high level) were 85.2, 45.7, and 11.4%, respectively, while no isolates were resistant to ampicillin, vancomycin and teicoplanin. The most prevalent resistance gene was *erm(B)* (97%), and any of the four genes encoding aminoglycoside modifying enzyme (AME) were detected in 99 isolates (47%). The AME gene *aac(6′)-Ie-aph(2”)-Ia* was detected in 46 isolates (21.9%) and was diverse in terms of IS*256*-flanking patterns, which were associated with resistance level to gentamicin. Tetracycline resistance was ascribable to *tet(M)* (61%) and *tet(L)* (38%), and mutations in the quinolone resistance-determining region of both GyrA and ParC were identified in 44% of isolates. Five isolates (2.4%) exhibited non-susceptibility to linezolide (MIC, 4 μg/mL), and harbored the oxazolidinone resistance gene *optrA*, which was located in a novel genetic cluster containing the phenicol exporter gene *fexA*. The *optrA*-positive isolates belonged to ST59, ST902, and ST917 (CC59), while common lineages of other multiple drug-resistant isolates were ST6, ST28, CC16, and CC116. The present study first revealed the prevalence of drug resistance determinants of *E. faecalis* and their genetic profiles in Bangladesh.

## 1. Introduction

*Enterococcus* is ubiquitously distributed in the environment and constitutes the normal flora of the intestinal tract in humans and animals. However, among this genus, particularly *Enterococcus faecalis* and *Enterococcus faecium* have been recognized as the common opportunistic pathogens implicated in urinary tract infections, wound/surgical site infections, and catheter-associated infections of the bloodstream [1]. *E. faecalis* is far more frequently isolated from clinical specimens than *E. faecium*, causing more intractable infectious disease, which is related to an increased level of drug resistance and the ability to form biofilm [2].

*E. faecalis* has an ability to acquire resistance to several antimicrobials such as aminoglycoside (high-level), penicillins, glycopeptides, quinolones, tetracyclines, and macrolides, via transposons, plasmids, or mutations, while it exhibits intrinsically reduced susceptibility to cephalosporins, aminoglycoside, lincosamide, trimethoprim-sulphamethoxazole [3]. Traditionally, resistance to penicillin, aminoglycoside (high-level), glycopeptide has been the major issue of clinical practice in the treatment of enterococcal infections. For such drug-resistant *E. faecalis* strains, represented by those with vancomycin resistance, newer compounds including linezolid, daptomycin, and tigecyclines have been used recently. Linezolid, a substance of oxazolidinone, has been a promising agent against infections caused by vancomycin-resistant enterococci (VRE), methicillin-resistant *S. aureus* (MRSA), and penicillin-resistant pneumococci since the turn of the twenty-first century [4]. However, during the past decade, resistance to oxazolidinones and daptomycin has been reported in many parts of the world, posing a concern for a serious limitation in the treatment of human infections caused by VRE and MRSA [5,6,7]. Linezolid resistance is attributable to mutations in the 23S rRNA and *rplC*/*rplD* encoding the 50S ribosomal proteins L3/L4, or the acquisition of *optrA* encoding an ATP-binding cassette (ABC)-F protein and *cfr* variant coding for 23S rRNA methyltransferase [5]. Daptomycin resistance is associated with increased cell wall thickening and other structural alterations of the cell wall caused by mutations in various genes responsible for call wall biosynthesis [5,6].

While aminoglycoside (gentamicin) combined with ampicillin has been the standard regimens of treatment for infective endocarditis due to enterococcus [8], recently designed regimens also include adaptomycin, tigecycline, and ceftaroline, together with ampicillin, gentamicin, or fosfomycin [2]. Moreover, the increased expression of tetracycline resistance determinants (*tet(L)*, *tet(M)*) was revealed to confer tigecycline resistance to *E. faecium* [5,9]. Accordingly, for the control of enterococcal infections, it is essential to understand the prevalence of resistance to conventional drugs as well as new compounds, and also their resistance mechanisms.

In Bangladesh, only limited information is available for the drug resistance of *Enterococcus* compared with *S. aureus* and Gram-negative bacteria [10]. *Enterococcus* was described as the second most frequent bacterial species from puerperal infections [11] and the third most common isolate from urinary tract infections [12]. In our previous study of puerperal infections, though the number of clinical isolates was low, *E. faecalis* was totally resistant to gentamycin, and approximately half of the isolates showed resistance to minocycline, erythromycin, and levofloxacin [11]. Similarly, high resistance rates to gentamicin and ciprofloxacin were observed for *E. faecalis* isolates from urinary tract infections [12,13], suggesting the spread of drug-resistant enterococcal strains in this country. However, genetic mechanisms of the resistance have not yet been studied. In the present study, we investigated the prevalence of resistance to clinically important antimicrobials and genetic mechanisms of the resistance for *E. faecalis* clinical isolates, to delineate the comprehensively drug resistant traits of this species in Bangladesh. We describe here the first identification of oxazolidinone resistance gene *optrA* in a novel *fexA–optrA* cluster in the country, in addition to characteristics of the genetic features of high-level gentamicin resistance gene, and other determinants of the conventional drug resistance of *E. faecalis*.

## 2. Materials and Methods

### 2.1. Bacterial Isolates

A total of 210 *E. faecalis* clinical isolates from the urine specimens of patients with urinary tract infections were analyzed. These isolates were collected in Mymensingh Medical College (MMC) hospital and Swadesh private hospital in Mymensingh, Bangladesh, consecutively, for a 15 month period starting from January 2018. The MMC hospital is only a tertiary referral medical center in the Mymensingh division, having 1000 beds, and the Swadesh private hospital is a 30 bedded private care facility. The age range of patients with a urinary tract infection was 1–80 years, while the sex ratio (male/female) was 1.3 (120/90). Only one isolate per patient was included in this study. Urine specimens were inoculated onto a chromogenic agar plate (HiCrome UTI Agar, HiMedia Laboratories), followed by incubation at 37 °C for 48 h aerobically. Bacterial colonies on the agar plates, which were morphologically assigned to *E. faecalis,* were picked up and further examined by Gram staining, catalase test, oxidase test and nitrate reduction test [4]. All the isolates were confirmed as *E. faecalis* by the PCR targeting PBP5 gene, with the use of primers and conditions reported previously [14]. For some isolates which could not be identified as *E. faecalis* by the PCR, the species was confirmed by the determination of the 16S rRNA gene sequence through direct sequencing with PCR product amplified by specific primers, as previously described [15]. Individual isolates were stored in Microbank (Pro-Lab Diagnostics, Richmond Hill, ON, Canada) at −80 °C, and were recovered when they were analyzed. This study was approved by the Institutional Review Board of MMC.

### 2.2. Antimicrobial Susceptibility Testing

Susceptibility to ampicillin (AMP), imipenem (IPM), gentamicin (GEN), minocycline (MIN), erythromycin (ERY), fosfomycin (FOF), levofloxacin (LVX), linezolid (LZD) teicoplanin (TEC), and vancomycin (VAN) was measured by broth microdilution test, using Dry Plate Eiken DP32 (Eiken, Tokyo, Japan). The minimum inhibitory concentration (MIC) of GEN was determined for all the isolates to judge for high-level resistance (MIC, ≥512 µg/mL). For the selected isolates, the MIC of kanamycin (KAN), chloramphenicol (CHL), florfenicol (FFC), daptomycin (DAP) and tedizolid (TDZ) were determined by broth microdilution method. Disk diffusion method was applied to tetracycline (TET) and nitrofurantoin (NIT). Susceptibility/resistance was judged according to the break points mentioned in the CLSI and EUCAST guidelines [16,17]. For CHL and FFC, the MIC breakpoints for susceptibility interpretation was done as described previously [18].

### 2.3. Detection of Drug Resistance Genes

The presence of following drug resistance genes was examined by uniplex or multiplex PCR assays by primers and conditions, as described previously [15,19,20,21,22]: beta-lactamase gene, *blaZ*; aminoglycoside modifying enzymes (AME) genes, *aac(6′)-Ie-aph(2″)-Ia*, *aph(3′)-IIIa*, *ant(6)-Ia*, *ant(4’)-Ia*, *aph(2”)-Id/Ie*, and *ant(9)-Ia*; macrolide resistance genes, *erm(A)*, *erm(B)*, *erm(C)*, *erm(T)*, *msr(A)*, and *msr(B)*; vancomycin resistance genes, *vanA*, *vanB*, *vanC*, *vanD*, *vanE*, and *vanG*; tetracycline resistance genes, *tet(L)*, *tet(M)*, *tet(K)*, *tet(O)*, *tet(S)*, *tet(T)*, and *tet(U)*; oxazolidinone and fenicol resistance gene, *optrA*. Nucleotide sequences of quinolone resistance-determining region (QRDR) of GyrA and ParC were determined by PCR and direct sequencing to detect mutations that are related to quinolone resistance [23].

### 2.4. Genetic Analysis of aac(6′)-Ie-aph(2″)-Ia

For all the isolates having *aac(6′)-Ie-aph(2″)-Ia* genes, IS*256*-flanking pattern (A, B, C or D) was assigned by PCR using the primers reported by Watanabe et al. [24]. For selected isolates with different IS*256*-flanking patterns and MIC to GEN, full-length gene sequences of *aac(6′)-Ie-aph(2″)-Ia* were determined directly from PCR products using the BigDye Terminator v. 3.1 Cycle Sequencing Kit (Applied Biosystems, Foster City, CA, USA) on an automated DNA sequencer (ABI PRISM 3100). The primers used for sequencing are shown in Table S1.

### 2.5. Genetic Determinants of Oxazolidinone and Daptomycin Resistance

Isolates exhibiting non-susceptibility to linezolid (MIC, ≥4 μg/mL) were further analyzed for the presences of *cfr*, *fexA*, *optrA* and mutation in 23S rRNA, L3- and L4- encoding genes as described previously [22,25,26,27]. The nucleotide sequence of the *fexA–optrA* gene cluster was determined for LZD-non-susceptible isolates by PCR and direct sequencing using the primers designed in this study (Table S1). In addition, for the isolates exhibiting a different MIC to daptomycin, the *pgsA* gene, which encodes phosphatidyl glycerophosphate synthase [6] was also sequenced with primers designed in this study (Table S1). The multiple alignment of nucleotide/amino acid sequences determined in the present study and those retrieved from the GenBank database was performed by Clustal Omega program (https://www.ebi.ac.uk/Tools/msa/clustalo/), which was also used for the calculation of sequence identity.

### 2.6. Multilocus Sequence Typing (MLST) 

For selected isolates having different drug resistance profiles and IS*256*-flanking pattern of *aac(6′)-Ie-aph(2″)-Ia*, the sequence type (ST) based on the MLST scheme [28] was identified using the web-based genotyping tool PubMLST (https://pubmlst.org/efaecalis/). The MLST data were further assigned to the clonal complex (CC) by BURST analysis available in the PubMLST website.

### 2.7. GenBank Accession Numbers

The nucleotide sequences of *fexA–optrA* and its cluster, *pgsA*, and *aac(6′)-Ie-aph(2″)-Ia* were deposited in the GenBank database under the accession numbers listed in Table S2.

## 3. Results

### 3.1. Prevalence of Antimicrobial Resistance and Resistance Determinants

The resistance rates to twelve antimicrobials and the detection rates of resistance determinants of 210 *E. faecalis* isolates are shown in Table 1. Resistance to ERY was found in 85.2% of all isolates, with *erm(B)* being highly prevalent (97.1%). Approximately 60% of isolates exhibited TET resistance, associated with *tet(M)* (61%) and/or *tet(L)* (38.1%). High-level resistance to gentamicin (GEN-HLR) was detected in 24 isolates (11.4%), while *aac(6′)-Ie-aph(2″)-Ia* was identified in 46 isolates (21.9%). Among all the *E. faecalis*, 99 isolates (47%) harbored any of the four AME genes, among which *aph(3′)-IIIa* was the most commonly detected (34.8%). Resistance to LVX was found in 45.7% of isolates, which had mostly double mutations, S84I in GyrA and S82I in ParC. Resistance to NIT was found in 10.5%, and no isolates were resistant to AMP, IPM, VAN, TEC, and FOF.

*optrA* and *fexA* were identified in five isolates (2.4%), which showed non-susceptibility to LZD (MIC, 4 μg/mL), and also resistance to CHL and FFC, showing a higher MIC than *optrA*-negative isolates, although the increased MIC of TDZ was not observed (Table S3). To the patients with these isolates, LZD had never been administered for treatment. In these isolates, the *cfr* gene was not detected, and no mutation was identified in the 23S rRNA gene (V domain) and L3- and L4-encoding genes (data not shown). Only a LZD-non-susceptible isolate (SJ116) showed non-susceptibility to DAP (MIC, 8 μg/mL). These five isolates had identical sequence of *pgsA*, of which the deduced amino acid sequences of the protein products were identical to those of *E. faecalis* strains S613 and R712, which were described as DAP-susceptible and resistant, respectively [6]. Single amino acid substitution, which was reported to be involved in DAP resistance in *S. aureus* [6], was not found in the five *optrA*-positive *E. faecalis* isolates (Figure S1).

### 3.2. Genetic Analysis of aac(6′)-Ie-aph(2″)-Ia

**Figure 1:**
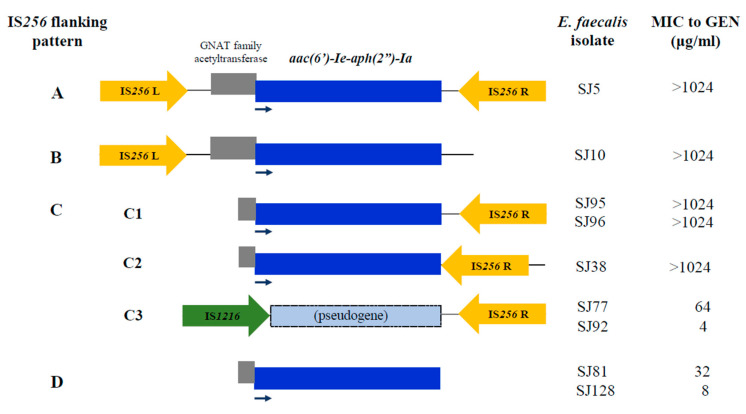
Schematic representation of the IS*256* flanking patterns of *aac(6′)-Ie-aph(2”)-Ia* (A-D) detected in *E. faecalis* isolates in the present study. A, Tn*4001*-like structure containing IS*256* (IS*256*-L and -R) at both ends; B-D, Tn*4001*-truncated structure lacking IS*256* at the 3′-end, 5′-end, and both ends, respectively. Intact open reading frame of *aac(6′)-Ie-aph(2”)-Ia* is shown as a blue box with an arrow indicating the transcription direction. The pseudogene in pattern C3 indicates the incomplete gene that lacks the 5′-end region including the start codon. *E. faecalis* isolate ID and MIC to GEN are shown on the right.

Forty-six *E. faecalis* isolates having *aac(6′)-Ie-aph(2″)-Ia* were classified into four IS*256*-flanking patterns A–D of this AME gene (9, 7, 7, and 23 isolates, respectively). All the isolates with pattern A having IS*256* at both sides, and pattern B having IS*256* at only upstream of *aac(6′)-Ie-aph(2″)-Ia* showed GEN-HLR (Figure 1, Table S4). In contrast, among pattern C, which has IS*256* at only downstream of this AME gene, and pattern D lacking IS*256* at both sides, GEN-HLR was observed in only a part of isolates; four among seven isolates with pattern C, 17% of pattern D isolates (4/23). Most of the isolates having pattern A- and B-*aac(6′)-Ie-aph(2″)-Ia* possessed also other AME genes (e.g., *aph(3′)-IIIa*), while solely *aac(6′)-Ie-aph(2″)-Ia* was carried mainly by pattern D isolates (Table S5).

Nucleotide sequences of *aac(6′)-Ie-aph(2″)-Ia* and its 5′- and 3′-end regions were determined for nine isolates representing the IS*256* flanking patterns A through D, and their genetic structures are shown schematically in Figure 1. The five isolates with pattern C examined were assigned to three different types (C1–C3). Although isolates showing GEN-HLR had C1 and C2 types which had intact *aac(6′)-Ie-aph(2″)-Ia* with IS*256* at different positions in its downstream, two GEN-susceptible isolates had a pseudogene of *aac(6′)-Ie-aph(2″)-Ia*, which was truncated by IS*1216* and lacked 5′-end region including start codon of this AME gene (C3 type). Two isolates of pattern D possessed intact *aac(6′)-Ie-aph(2″)-Ia*, despite showing non-GEN-HLR.

### 3.3. ST of Isolates with Different Characteristics

ST was identified for 39 *E. faecalis* isolates with different profiles of drug resistance and resistance genes, including those with different IS*256*-flanking patterns of *aac(6′)-Ie-aph(2″)-Ia* and those with *fexA–optrA* (Table 2). Isolates showing GEN-HLR were generally resistant to multiple classes of antimicrobials (macrolide, quinolone, tetracycline) and mostly belonged to ST6, ST28, and STs of CC28 and CC116. In contrast, isolates with pattern D-*aac(6′)-Ie-aph(2″)-Ia* were resistant to lesser number of the drugs examined and mostly assigned to CC16. Isolates without *aac(6′)-Ie-aph(2″)-Ia* and *optrA* had less resistance determinants showing resistance to less antimicrobials, and included the isolates of ST28 and its relevant STs (ST945, ST919). Five isolates having *fexA–optrA* were classified into ST59 (two isolates), ST902 (triple-locus variant of ST21, two isolates), and ST917 (single-locus variant of ST59, one isolate). Although these isolates had similar profile of resistance genes showing multiple drug resistance, CC59 isolates were resistant to LVX associated with QRDR mutations in GyrA and ParC, unlike ST902 isolates.

### 3.4. Genetic Background of fexA and optrA

The five isolates with non-susceptibility to LZD were analyzed for the *fexA–optrA* cluster, which comprises *fexA*, a short open reading frame, and *optrA*, with the same orientation. Nucleotide sequences of the *fexA–optrA* cluster from the five isolates were identical to that reported for *E. faecalis* strain 743,142 and 981,649 (Taiwan, clinical isolates) plasmids (GenBank accession nos. MF443377 and MF443386, respectively) by BLAST search. The deduced amino acid sequences of *optrA* (655 amino acids) of the five *E. faecalis* isolates have three divergent amino acids (K3E, Y176D, G393D) compared with the OptrA prototype in *E. faecalis* strain E349 (GenBank accession no. KP399637) [22], which corresponds to the “EDD variant” [29]. FexA (475 amino acids) of Bangladeshi isolates was different from that of strain E349 by four amino acids (Figure S2). When sequences of this gene of *E. faecalis*, and other enterococcal species and staphylococcus were retrieved from GenBank database, at least ten variants of FexA were identified, having divergent amino acids at 15 positions with 97.7–99.8% identity (Figure S2).

**Figure 2:**
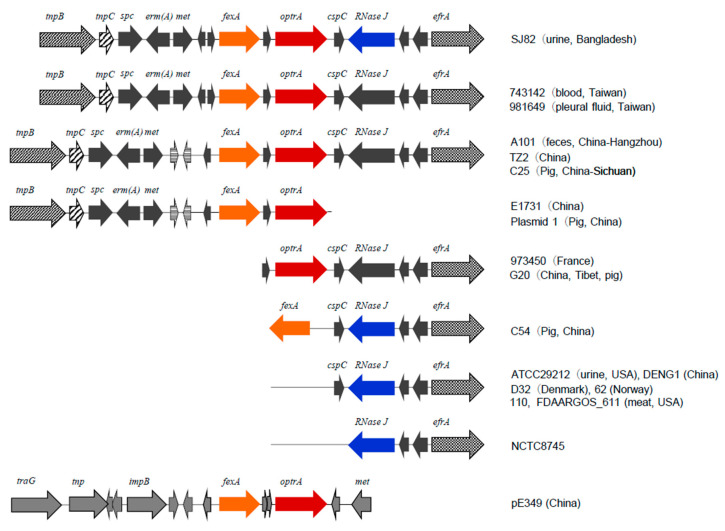
Schematic representation of the genetic background of *optrA* in the *E. faecalis* strain SJ82 (uppermost) and the genetic organization or components similar to that of SJ82 in other strains reported previously [30] or available in GenBank database. Prototype of the *fexA–optrA* cluster in the pE349 of *E. faecalis* strain E349 [22] is shown at the bottom. Arrows indicate the transcription direction of genes. Arrows of *RNase J* are shown in black and blue, representing different sequences. Gene names are shown above arrows, and the strain names are indicated on the right.

Strain SJ82 was further analyzed for the broader region containing *fexA–optrA* cluster (Figure 2) and compared with this region, reported for various strains previously [30]. This region of SJ82 contained *tnpB*, *tnpC*, *spc*, *erm(A)* upstream from *fexA*, having an identical sequence to those in the *E. faecalis* strains 743,142 and 981,649. Although similar genetic structure is seen in also *E. faecalis* strain TZ2 and E1731 (China), sequences of the connecting portion between *fexA* and *erm(A)* were different from SJ82. In the downstream of *optrA*, three genes (*cspC*, *RNase J*, and *efrA*) are located as seen in *E. faecalis* strains 981,649 (Taiwan), TZ2 (China), 973,450 (France), C54 (China), D32 (Denmark), and NCTC8745 [30]. The nucleotide sequence of *RNase J* was identical to that of strains C54, D32, and NCTC8745, while it was slightly different from strains 981,649 and 743,142 (sequence identity 98.9–99.2%) with divergent positions being dispersed in this gene (Figure S3). The genetic organization of the *fexA–optrA* cluster was quite distinct from that of the prototype of *optrA* in pE349 (GenBank accession no. KP399637) [22]. As a whole genetic organization of the *fexA–optrA* cluster region of SJ82 was similar to that of the *E. faecalis* strain 981649, except for *RNase J* which was identical to that of other strains (e.g., C54).

## 4. Discussion

The present study first characterized *E. faecalis* from urinary tract infections in Bangladesh for the situation of antimicrobial susceptibility and genetic factors related to drug resistance. While resistance to penicillins and glycopeptides was absent, higher resistance rates were noted for ERY, TET, and LVX. The prevalence of ERY resistance (85.2%) was higher than in our previous study of puerperal infections in Bangladesh [11], and comparable to that reported for blood isolates in the US and Europe [31], and hospital isolates in the middle East [32]. The resistance rate to LVX (45.7%) was similar to that in our previous studies in Bangladesh [11] and the Middle East [32], but appears to be higher than the global average [33]. TET resistance rate was much less prevalent than in the US and Europe [31]. The susceptibility rate of *E. faecalis* to nitrofurantoin, which is commonly prescribed for urinary tract infections, was higher in our study (89.5%) than in previous reports in Bangladesh and India (78–86%) [12,13,34].

The prevalence of GEN-HLR in the present study (11.4%) was substantially lower than in recent reports from India, the Middle East, Australia, and Japan (22–55%) [32,34,35,36,37]. However, *aac(6′)-Ie-aph(2″)-Ia*, which is responsible for GEN-HLR, was more prevalent (21.9%) than the phenotypically detected GEN-HLR. This discordance is considered to be in part related to diversity in IS*256*-flanking patterns and also the genetic alteration of this gene, as observed in our present study. Although GEN-HLR was found in the isolates with pattern A and B, a portion of pattern C and D isolates was not highly resistant to GEN. The presence of IS*256* at both sides of *aac(6′)-Ie-aph(2″)-Ia* (pattern A), which represents Tn*4001* [38], is relevant to GEN-HLR via elevated transcription of the AME gene due to the presence of IS*256* [39]. In contrast, *aac(6′)-Ie-aph(2″)-Ia* might not be transcribed efficiently in most of pattern D isolates which lacked adjacent IS*256*, moreover, this AME is unlikely to be produced in pattern C isolates having a pseudogene lacking a start codon of this gene. In the present study, pattern D was the most frequent, and accounted for half of the isolates harboring *aac(6′)-Ie-aph(2″)-Ia*. Similarly, the dominance of pattern C and D representing truncated transposon structures of this gene was observed among the clinical isolates in other countries [24,40,41]. In addition, some variations in pattern C were also found in other reports [40,41]. It is suggested that intact Tn*4001*-like transposon containing this AME gene might have evolved through recombination events [41], and the resultant variants of this AME gene associated with non-GEN-HLR might have been persisting probably due to less exposure of GEN to *E. faecalis* in current antibiotic therapy.

It was remarkable that oxazolidinone resistance-determinant *optrA* was identified in five *E. faecalis* isolates showing non-susceptibility to LZD, although these isolates were derived from urinary tract infections without the use of this antimicrobial for treatment. The protein product of *optrA* belongs to the ATP-binding cassette (ABC)-F protein superfamily that mediates resistance through ribosomal protection [42], which is more of a common cause of oxazolidinone resistance in enterococci [30,43,44], compared with other genetic determinants *cfr* or mutations in 23S rRNA and ribosomal proteins. LZD-resistant *E. faecalis* have been reported worldwide, particularly more frequently in China [7]. Although the non-susceptibility rate to the LZD of enterococci was reported as <1% at the global level [7,30], the prevalence of LZD-resistance/non-susceptibility or *optrA* is evidently higher in China (1–4%) [22,44,45,46,47], with a higher rate in *E. faecalis* than in *E. faecium*. Moreover, in China, the increase in *optrA*-positive rate in enterococci was documented from 2004 (0.4%) to 2014 (3.9%) [46], and *E. faecalis* harboring *optrA* was isolated from the stool samples of healthy adults and children with a carriage rate of 3.5% [29]. The present detection rate of *optrA* in *E. faecalis* in Bangladesh (2.4%) may be comparable to those reported in China, implying relatively high prevalence among clinical isolates in this country, and may suggest also the potential distribution among the healthy population since LZD has been rarely used. The occurrence of *optrA*-positive *E. faecalis* was reported also in patients having no history of LZD treatment in Korea [48]. Because the five LZD-non-susceptible isolates in our study exhibited resistance to multiple drugs including NIT, it is possible that these isolates might have occurred by selection with ordinary antimicrobial treatment.

Globally distributed *optrA*-positive *E. faecalis* reported to date belong to various STs, including major types ST16, ST116, ST256, ST476, ST480, ST585, with ST16 being dominant [22,45,47,49,50,51,52]. The five isolates with *optrA* in Bangladesh belonged to ST59, ST902 and ST917, among which ST902 is triple-locus variant of ST21, and ST917 is single-locus variant of ST59 (CC59). This indicates that the Bangladeshi isolates comprise two lineages, ST21-like (ST902) and CC59 clones, which had been revealed to be phylogenetically distinct [52]. ST21 and/or ST59 were reported as minor lineages in China [22,43,45], Malaysia [30], and Germany [49].

The five *optrA*-positive *E. faecalis* isolates had an “EDD” variant which represents one of the 25 types of OptrA amino acid sequence [29]. The isolates with the “EDD” variant in China showed intermediate resistance to LZD (4 μg/mL), as observed in our present study, and accounted for 24% and 13% of the *optrA*-positive isolates from healthy humans and clinical isolates, respectively [29,46]. In contrast, a higher MIC to LZD (≥8 μg/mL) was evident for the isolates with wild-type *optrA* and some variants, e.g., “RDK” [22,29]. Although *E. faecalis,* with intermediate resistance to LZD from urinary tract infections, as those detected in our study, may not cause issues in treatment, such isolates are suggested to increase the potential risk for opportunistic cross-infections in a healthcare setting.

Various genetic backgrounds containing *optrA* have been documented in the context of plasmid or chromosome of enterococci [30]. The *fexA–optrA* cluster, which was detected in *E. faecalis* in Bangladesh, is one of the major genetic structures in clinical isolates from humans, including the prototype strain E349 [29], as well as isolates from animals (chicken, pig) and retail meat [18,30,53]. *tnpB* and *tnpC* were associated with the *fexA–optrA* region of strain SJ82, which suggested that this *optrA* cluster may be located on a Tn*554* family transposon (e.g., Tn*6674*) as described for that containing *optrA* in *E. faecalis* [54]. In the present study, the *fexA–optrA* cluster and its upstream region including the *erm(A)* and *spc* of the Bangladeshi strain SJ82 were found to be genetically distinct from those of E349, but identical to those of *E. faecalis* clinical isolates in Taiwan, and highly similar to those of *E. faecalis* from humans and animals in China [30]. In contrast, the *NRase J* gene located downstream from *optrA* was identical to other strains in China and other countries [30]. The identification of such a novel genetic background of *optrA* in strain SJ82 suggests the occurrence of recombinations in *optrA*-containing regions among *E. faecalis* distributed in humans and/or animals in Asia. Since this *optrA* region contains other resistance genes (*erm(A)*, *spc*, and *fexA*), it is possible that the selective dissemination of *optrA* may be caused by the use of macrolides and spectinomycin to humans, or florfenicol to animals. Among the five isolates with *optrA*, an isolate SJ116 exhibited non-susceptibility to DAP. In the present study, no mutation was detected in the *pgsA* of strains SJ116 and also previously reported DAP-non-susceptible strains, although mutation in *pgsA* was involved in DAP resistance in *S. aureus* and *Corynebacterium striatum* [6]. Although various genes are revealed to be implicated in DAP resistance in enterococci [5], it was suggested that a mutation in *pgsA* may not mediate the primary role in decreased susceptibility to DAP.

## 5. Conclusions

The present study on *E. faecalis* clinical isolates in Bangladesh revealed considerably high resistance rates to ERY and LVX, while a low rate of GEN-HLR and none with glycopeptide and penicillin resistance among them. LZD-non-susceptible isolates harboring *optrA* were first identified in this country, indicating the need for further epidemiological investigation to determine its prevalence and clinical significance. 

## Figures and Tables

**Table 1:** Antimicrobial resistance and resistance determinants of *E. faecalis* clinical isolates in this study.

Antimicrobials ^1^/Resistance Determinants ^2^		Number of Resistant Isolates/Isolates with Resistant Determinant (%) (*n* = 210)
Antimicrobial agents		
	AMP	0 (0)
	IPM	0 (0)
	GEN-HLR	24 (11.4)
	VAN	0 (0)
	TEC	0 (0)
	ERY	179 (85.2)
	TET	125 (59.5)
	MIN	17 (8.1)
	NIT	22 (10.5)
	FOF	0 (0)
	LVX	96 (45.7)
	LZD	5 ^4^ (2.4)
Resistance gene/determinant		
(Aminoglycoside)	*aac(6′)-Ie-aph(2″)-Ia*	46 (21.9)
	*aph(3′)-IIIa*	73 (34.8)
	*ant(6)-Ia*	18 (8.6)
	*ant(9)-Ia*	8 (3.8)
(Macrolide)	*erm(A)*	1 (0.5)
	*erm(B)*	204 (97.1)
(Tetracycline)	*tet(L)*	80 (38.1)
	*tet(M)*	128 (61.0)
(Oxazolidinone)	*fexA-optrA*	5 (2.4)
(QRDR ^3^ mutation)	GyrA: S 84 I and ParC: S 82 I	93 (44.3)
	GyrA: S 84 I	3 (1.4)

^1^ Abbreviations: AMP, Ampicillin; ERY, Erythromycin; FOF, Fosfomycin; GEN-HLR, Gentamicin, high-level resistance; IPM, Imipenem; LVX, Levofloxacin; LZD, Linezolid; MIN, Minocycline; NIT, nitrofurantoin; TEC, Teicoplanin; TET, Tetracycline; VAN, Vancomycin. ^2^ Following genes were not detected in any isolate: *blaZ*, *erm(C)*, *erm(T)*, *msr(A)*, *msr(B)*, *tet(K)*, *tet(O)*, *tet(T)*, *tet(U)*, *vanA*, *vanB*, *vanC*, *vanD*, *vanE* and *vanG*. ^3^ QRDR, quinolone resistance determining region. ^4^ Number of isolates showing non-susceptibility to LZD (MIC, 4 μg/mL).

**Table 2:** Genotypes, antimicrobial resistance profile and resistance genes/genetic determinants of selected *E.faecalis* isolates (*n* = 39).

Isolate ID	Age/Sex of Patient	Antimicrobial Resistance Pattern ^1^	Drug Resistance Genes ^2^	MIC (μg/mL) of GEN	IS256 Flanking Pattern (*aac-(6′)-Ie-aph(2″)-Ia*)	MIC (μg/mL) of LVX	QRDR Mutation ^3^		Sequence Type ^4^ (MLST)	Clonal Complex (CC), ST Variant
							GyrA	ParC		
SJ5	70/M	ERY, GEN-HLR, KAN, LVX, TET, NIT	*aac(6′)-Ie-aph(2″)-Ia, aph(3′)-IIIa, ant(6)-Ia, erm(B), tet(M)*	>1024	A	64	S 84 I	S 82 I	ST28	CC28
SJ42	3/F	ERY, GEN-HLR, KAN, LVX, TET, NIT	*aac(6′)-Ie-aph(2″)-Ia, aph(3′)-IIIa, ant(6)-Ia, erm(B), tet(M)*	>1024	A	64	S 84 I	S 82 I	ST28	CC28
SJ94	45/M	ERY, GEN-HLR, LVX, TET	*aac(6′)-Ie-aph(2″)-Ia, ant(6)-Ia, erm(B), tet(M)*	>1024	A	32	S 84 I	S 82 I	ST28	CC28
SJ32	30/F	ERY, GEN-HLR, KAN, LVX, TET, NIT	*aac(6′)-Ie-aph(2″)-Ia, aph(3′)-IIIa, erm(B), tet(M)*	>1024	A	128	S 84 I	S 82 I	ST28	CC28
SJ238	38/M	ERY, GEN-HLR, LVX, TET, KAN	*aac(6′)-Ie-aph(2″)-Ia, aph(3′)-IIIa, erm(B), tet(L), tet(M)*	>1024	A	32	S 84 I	S 82 I	ST946	CC116
SJ125	40/M	ERY, GEN-HLR, LVX, TET, MIN, KAN	*aac(6′)-Ie-aph(2″)-Ia, aph(3′)-IIIa, ant(6)-Ia, ant(9)-Ia, erm(A), erm(B), tet(L), tet(M)*	>1024	A	32	S 84 I	S 82 I	ST6	CC6
SJ204	3.5/F	ERY, GEN-HLR, KAN, LVX, TET, NIT	*aac(6′)-Ie-aph(2″)-Ia, aph(3′)-IIIa, ant(6)-Ia, erm(B), tet(M)*	>1024	A	32	S 84 I	S 82 I	ST6	CC6
SJ40	5/F	ERY, GEN-HLR, LVX, TET, MIN, NIT	*aac(6′)-Ie-aph(2″)-Ia, erm(B), tet(M)*	>1024	A	64	S 84 I	S 82 I	ST6	CC6
SJ127	18/M	ERY, GEN-HLR, KAN, LVX, TET, MIN, NIT	*aac(6′)-Ie-aph(2″)-Ia, aph(3′)-IIIa, ant(6)-Ia, erm(B), tet(M)*	>1024	A	32	S 84 I	S 82 I	ST6	CC6
SJ208	20/F	ERY, GEN-HLR, KAN, TET, NIT	*aac(6′)-Ie-aph(2″)-Ia, aph(3′)-IIIa, ant(6)-Ia, erm(B), tet(M)*	>1024	B	< 2	NM	NM	ST363	CC16
SJ207	55/M	ERY, GEN-HLR, KAN, LVX, TET	*aac(6′)-Ie-aph(2″)-Ia, aph(3′)-IIIa, erm(B), tet(L), tet (M)*	>1024	B	64	S 84 I	S 82 I	ST28	CC28
SJ3	40/F	ERY, GEN-HLR, KAN, LVX, TET	*aac(6′)-Ie-aph(2″)-Ia, aph(3′)-IIIa, ant(6)-Ia, erm(B), tet(M)*	>1024	B	128	S 84 I	S 82 I	ST28	CC28
SJ10	2/M	ERY, GEN-HLR, KAN, LVX, TET, NIT	*aac(6′)-Ie-aph(2″)-Ia, aph(3′)-IIIa, ant(6)-Ia, erm(B), tet(M)*	>1024	B	64	S 84 I	S 82 I	ST6	CC6
SJ8	47/F	ERY, GEN-HLR, KAN, LVX, TET, NIT	*aac(6′)-Ie-aph(2″)-Ia, aph(3′)-IIIa, ant(6)-Ia, erm(B), tet(M)*	>1024	B	128	S 84 I	S 82 I	ST28	CC28
SJ11	22/F	ERY, GEN-HLR, KAN, LVX, TET, NIT	*aac(6′)-Ie-aph(2″)-Ia, aph(3′)-IIIa, ant(6)-Ia, erm(B), tet(M)*	>1024	B	64	S 84 I	S 82 I	ST965 *	ST919 SLV
SJ13	2/M	ERY, GEN-HLR, KAN, LVX, TET	*aac(6′)-Ie-aph(2″)-Ia, aph(3′)-IIIa, ant(6)-Ia, erm(B), tet(M)*	>1024	B	64	S 84 I	S 82 I	ST966 *	CC28
SJ38	5/F	ERY, GEN-HLR, KAN, LVX, TET, MIN	*aac(6′)-Ie-aph(2″)-Ia, aph(3′)-IIIa, ant(6)-Ia, erm(B), tet(L), tet(M)*	>1024	C	16	S 84 I	S 82 I	ST28	CC28
SJ77	65/M	TET	*aac(6′)-Ie-aph(2″)-Ia, erm(B), tet(L), tet(M)*	64	C	< 2	NM	NM	ST947 *	CC116
SJ92	33/F	ERY, KAN, LVX, TET	*aac(6′)-Ie-aph(2″)-Ia, aph(3′)-IIIa, erm(B), tet(L), tet(M)*	4	C	16	S 84 I	S 82 I	ST947 *	CC116
SJ95	50/M	ERY, GEN-HLR, KAN, LVX, TET	*aac(6′)-Ie-aph(2″)-Ia, aph(3′)-IIIa, erm(B), tet(L), tet(M)*	>1024	C	32	S 84 I	S 82 I	ST947 *	CC116
SJ96	32/F	ERY, GEN-HLR, KAN, LVX, TET, MIN	*aac(6′)-Ie-aph(2″)-Ia, aph(3′)-IIIa, erm(B), tet(L), tet(M)*	>1024	C	16	S 84 I	S 82 I	ST947 *	CC116
SJ128	28/F	ERY, TET	*aac(6′)-Ie-aph(2″)-Ia, erm(B), tet(M)*	128	D	< 2	NM	NM	ST16	CC16
SJ132	30/F	ERY, TET	*aac(6′)-Ie-aph(2″)-Ia, erm(B), tet(M)*	128	D	< 2	NM	NM	ST16	CC16
SJ134	30/M	ERY, TET	*aac(6′)-Ie-aph(2″)-Ia, erm(B), tet(M)*	64	D	< 2	NM	NM	ST16	CC16
SJ31	1/F	ERY, GEN-HLR, KAN, LVX, TET	*aac(6′)-Ie-aph(2″)-Ia, aph(3′)-IIIa, ant(6)-Ia, erm(B), tet(M)*	>1024	D	32	S 84 I	NM	ST415	CC941
SJ81	40/M	ERY, LVX, TET	*aac(6′)-Ie-aph(2″)-Ia, erm(B), tet(M)*	32	D	< 2	NM	NM	ST16	CC16
SJ126	30/F	ERY, LVX, TET	*aac(6′)-Ie-aph(2″)-Ia, erm(B), tet(M)*	128	D	< 2	NM	NM	ST818	CC16
SJ218	11/F	LVX, TET, MIN	*ant(9)-Ia, erm(B), erm(B), tet(L), tet(M)*	8		16	S 84 I	NM	ST945 *	CC28
SJ71	7/F	ERY, KAN, TET, MIN	*aph(3′)-IIIa, ant(9)-Ia, erm(B), tet(L), tet(M)*	8		< 2	NM	NM	ST21	CC21
SJ28	55/F	ERY, KAN, LVX, TET	*aph(3′)-IIIa, ant(6)-Ia, erm(B), tet(M)*	16		64	S 84 I	S 82 I	ST28	CC28
SJ69	27/F	ERY, KAN, TET	*aph(3′)-IIIa, ant(9)-Ia, erm(B), tet(M)*	8		< 2	NM	NM	ST506	CC100
SJ80	50/M	ERY	*erm(B)*	8		< 2	NM	NM	ST919 *	ST28 TLV
SJ148	33/F	ERY	*erm(B), tet(L)*	4		< 2	NM	NM	ST919 *	ST28 TLV
SJ52	24/F	ERY, TET	*erm(B), tet(L), tet(M)*	2		< 2	NM	NM	ST919 *	ST28 TLV
SJ87	20/M	ERY, KAN, LVX, TET, NIT, LZD	*aph(3′)-IIIa, ant(9)-Ia, erm(B), tet(L), tet(M), fexA-optrA*	8		8	S 84 I	S 82 I	ST59	CC59
SJ88	18/M	ERY, KAN, LVX, TET, MIN, NIT, LZD	*aph(3′)-IIIa, ant(9)-Ia, erm(B), tet(L), tet(M), fexA-optrA*	4		8	S 84 I	S 82 I	ST59	CC59
SJ82	25/F	ERY, KAN, TET, MIN, NIT, LZD	*aph(3′)-IIIa, ant(9)-Ia, erm(B), tet(L), tet(M), fexA-optrA*	8		< 2	NM	NM	ST902	ST21 TLV
SJ117	50/M	ERY, KAN, TET, MIN, NIT, LZD	*aph(3′)-IIIa, ant(9)-Ia, erm(B), tet(L), tet(M), fexA-optrA*	4		< 2	NM	NM	ST902	ST21 TLV
SJ116	28/F	ERY, KAN, LVX, TET, MIN, NIT, LZD, DAP	*aph(3′)-IIIa, ant(9)-Ia, erm(B), tet(L), tet(M), fexA-optrA*	8		8	S 84 I	S 82 I	ST917 *	CC59

^1^ Abbreviations: AMP, Ampicillin; DAP, daptomycin; ERY, Erythromycin; FOF, Fosfomycin; HL-GEN, Gentamicin, high level resistance; IPM, Imipenem; KAN, kanamycin; LVX, Levofloxacin; LZD, Linezolid; MIN, Minocycline; TEC, Teicoplanin; TET, Tetracycline; VAN, Vancomycin. LZD and DAP indicate non-susceptibility (MIC, 4 μg/mL and 8 μg/mL, respectively). Five LZD-non-susceptible isolates are shown at the bottom. None of isolates showed resistance to AMP, IMP, TEC, VAN, and FOF. ^2^ All isolates were negative for *blaZ*, *erm(C)*, *erm(T)*, *msr(A)*, *msr(B)*, *tet(K)*, *tet(O)*, *tet(T)*, *tet(U)*, *vanA*, *vanB*, *vanC*, *vanD*, *vanE* and *vanG*. ^3^ QRDR, quinolone resistance determining region; NM, no mutation detected. ^4^ Novel ST detected in this study is shown with asterisk (*). SLV, single-locus variant; TLV, triple-locus variant.

## Supplementary Materials

The following are available online at https://www.mdpi.com/2076-2607/8/8/1240/s1, **Figure S1**: Amino acid sequence alignment of PgsA (phosphatidylglycerol synthase) among *E. faecalis* strains, **Figure S2**: Amino acid sequence alignment of chloramphenicol/florfenicol efflux MFS (major facilitator superfamily) transporter FexA of *E. faecalis* strains, **Figure S3**: Alignment of nucleotide (a) and amino acid (b) sequences of RNase J family beta-CASP ribonuclease gene of *E. faecalis* strains SJ82, 981649 and 743142, **Table S1**: Primers used for the analysis of oxazolidinone resistance determinants, *pgsA*, and *aac(6′)-Ie-aph(2")-Ia*, **Table S2**: GenBank accession numbers assigned to *fexA–optrA* (cluster), *pgsA*, and *aac(6′)-Ie-aph(2")-Ia* detected in the *E. faecalis* clinical isolates in the present study, **Table S3**: Additional information of the antimicrobial susceptibility of five *optrA*-positive isolates and two *optrA*-negative isolates, **Table S4**: IS*256* flanking pattern of *aac(6′)-Ie-aph(2″)-Ia* and MIC to GEN, **Table S5**: AME gene profile and IS*256* flanking pattern of *aac(6′)-Ie-aph(2″)-Ia*.
