# Supplementary material for: Drug Resistance Determinants in Clinical Isolates of Enterococcus faecalis in Bangladesh: Identification of Oxazolidinone Resistance Gene optrA in ST59 and ST902 Lineages
Source: Microorganisms. 2020 Aug 14;8(8):1240. doi: 10.3390/microorganisms8081240 (PMC7463919; doi:10.3390/microorganisms8081240)
Supplement: Supplementary file 1 [file microorganisms-08-01240-s001.zip › Supplementary Material SRoy/FigS1-PgsA SRoy.docx]

(a)

SJ82 MNLPNKLTVLRIFMIPIFIIIVSVPMDWGTISFGDTTLAVTQLVGAIIFAVASFTDWLDG 60

SJ87 MNLPNKLTVLRIFMIPIFIIIVSVPMDWGTISFGDTTLAVTQLVGAIIFAVASFTDWLDG 60

SJ88 MNLPNKLTVLRIFMIPIFIIIVSVPMDWGTISFGDTTLAVTQLVGAIIFAVASFTDWLDG 60

SJ116 MNLPNKLTVLRIFMIPIFIIIVSVPMDWGTISFGDTTLAVTQLVGAIIFAVASFTDWLDG 60

SJ117 MNLPNKLTVLRIFMIPIFIIIVSVPMDWGTISFGDTTLAVTQLVGAIIFAVASFTDWLDG 60

R712 MNLPNKLTVLRIFMIPIFIIIVSVPMDWGTISFGDTTLAVTQLVGAIIFAVASFTDWLDG 60

S613 MNLPNKLTVLRIFMIPIFIIIVSVPMDWGTISFGDTTLAVTQLVGAIIFAVASFTDWLDG 60

************************************************************

SJ82 KIARAQGLVTNFGKFADPLADKMLVMTAFIVLVGQGKVPAWIVAIIVCRELAVTGLRLLL 120

SJ87 KIARAQGLVTNFGKFADPLADKMLVMTAFIVLVGQGKVPAWIVAIIVCRELAVTGLRLLL 120

SJ88 KIARAQGLVTNFGKFADPLADKMLVMTAFIVLVGQGKVPAWIVAIIVCRELAVTGLRLLL 120

SJ116 KIARAQGLVTNFGKFADPLADKMLVMTAFIVLVGQGKVPAWIVAIIVCRELAVTGLRLLL 120

SJ117 KIARAQGLVTNFGKFADPLADKMLVMTAFIVLVGQGKVPAWIVAIIVCRELAVTGLRLLL 120

R712 KIARAQGLVTNFGKFADPLADKMLVMTAFIVLVGQGKVPAWIVAIIVCRELAVTGLRLLL 120

S613 KIARAQGLVTNFGKFADPLADKMLVMTAFIVLVGQGKVPAWIVAIIVCRELAVTGLRLLL 120

************************************************************

SJ82 VEHGEVMAAAWPGKVKTATQMVAIILLFINNIPFSALHLPLDQIMLYACLIFTIYSGVDY 180

SJ87 VEHGEVMAAAWPGKVKTATQMVAIILLFINNIPFSALHLPLDQIMLYACLIFTIYSGVDY 180

SJ88 VEHGEVMAAAWPGKVKTATQMVAIILLFINNIPFSALHLPLDQIMLYACLIFTIYSGVDY 180

SJ116 VEHGEVMAAAWPGKVKTATQMVAIILLFINNIPFSALHLPLDQIMLYACLIFTIYSGVDY 180

SJ117 VEHGEVMAAAWPGKVKTATQMVAIILLFINNIPFSALHLPLDQIMLYACLIFTIYSGVDY 180

R712 VEHGEVMAAAWPGKVKTATQMVAIILLFINNIPFSALHLPLDQIMLYACLIFTIYSGVDY 180

S613 VEHGEVMAAAWPGKVKTATQMVAIILLFINNIPFSALHLPLDQIMLYACLIFTIYSGVDY 180

************************************************************

SJ82 FAKNKDVFKGSM 192

SJ87 FAKNKDVFKGSM 192

SJ88 FAKNKDVFKGSM 192

SJ116 FAKNKDVFKGSM 192

SJ117 FAKNKDVFKGSM 192

R712 FAKNKDVFKGSM 192

S613 FAKNKDVFKGSM 192

************

(b)

SJ116 MNLPNKLTVLRIFMIPIFIIIVSVPMDWGTISF-GDTTLAVTQLVGAIIFAVASFTDWLD 59

N315 MNIPNQITVFRVVLIPVFILFALVDFGFGNVSFLGGYEIRIELLISGFIFILASLSDFVD 60

**:**::**:*:.:**:**::. * :.:*.:** *. : : *:..:** :**::*::*

SJ116 GKIARAQGLVTNFGKFADPLADKMLVMTAFIVLVGQGKVPAWIVAIIVCRELAVTGLRLL 119

N315 GYLARKWNLVTNMGKFLDPLADKLLVASALIVLVQLGLTNSVVAIIIIAREFAVTGLRLL 120

* :** .****:*** ******:** :*:**** * . : :. **:.**:********

▼

SJ116 LVEHGEVMAAAWPGKVKTATQMVAIILLFINNIPFSALHLPLDQIMLYACLIFTIYSGVD 179

N315 QIEQGFVSAAGQLGKIKTAVTMVAITWLLLGDPLATLIGLSLGQILLYIGVIFTILSGIE 180

:*:* * **. **:***. **** *::.: : : * *.**:** :**** **::

SJ116 YFAKNKDVFKGSM 192

N315 YFYKGRDVFKQK- 192

** *.:**** .

**Figure S1.** Amino acid sequence alignment of PgsA (phosphatidylglycerol synthase) among *E. faecalis* strains (a), and DAP-non-susceptible strain SJ116 and *S. aureus* strain N315 (b). Arrowhead indicates amino acid position 135 of N315 (equivalent position 134 in *E. faecalis*) where substitution of lysin (in yellow) to glutamic acid is implicated in DAP resistance in *S. aureus* [6]. Asterisk indicates identical amino acid. Dash denotes gap.
