# Supplementary material for: Drug Resistance Determinants in Clinical Isolates of Enterococcus faecalis in Bangladesh: Identification of Oxazolidinone Resistance Gene optrA in ST59 and ST902 Lineages
Source: Microorganisms. 2020 Aug 14;8(8):1240. doi: 10.3390/microorganisms8081240 (PMC7463919; doi:10.3390/microorganisms8081240)
Supplement: Supplementary file 1 [file microorganisms-08-01240-s001.zip › Supplementary Material SRoy/FigS2-FexA SRoy.docx]

**(a)**

E.faecalis-E349 MKKDSKSKEMIQSEKRGSTRLLMMVLSLSVLVGSITADSVNPVLPLISKALEASKSQVSW 60

E.faecalis-SJ82 MKKDSKSKEMIQSEKRGSTRLLMMVLSLSVLVGAITSDIVNPVLPLISKDLEASKSQVSW 60

*********************************:**:* ********** **********

E.faecalis-E349 IVSGIALVLAIGVPIYGRISDFFELRKLYIFAIMILASGSLLCAIAPNLPLLVLGRMVQG 120

E.faecalis-SJ82 IVSGIALVLAIGVPIYGRISDFFELRKLYIFAIMILASGSLLCAIAPNLPLLVLGRMVQG 120

************************************************************

E.faecalis-E349 AGMSAIPVLSVIAISKVFPQGKRGGALGIIAGSIGVGTAAGPIFGGVVGQYLGWNALFWF 180

E.faecalis-SJ82 AGMSAIPVLSVIAISKVFPQGKRGGALGIIAGSIGVGTAAGPIFGGVVGQYLGWNALFWF 180

************************************************************

E.faecalis-E349 TFLLAIMIVIGAYYALPTIKPAESVGSNKNFDFIGGLFLGLTVGLLLFGITQGETSGFSS 240

E.faecalis-SJ82 TFLLAIMIVIGAYYALPTIKPAESVGSNKNFDFIGGLFLGLTVGLLLFGITQGETSGFSS 240

************************************************************

E.faecalis-E349 FSSLTSLIGSVVALVGFIWRIVTAENPFVPPVLFNNKDYVNTVIIAFFSMFAYFAVLVFV 300

E.faecalis-SJ82 FSSLTSLIGSVVALVGFIWRIVTAENPFVPPVLFNNKDYVNTVIIAFFSMFAYFAVLVFV 300

************************************************************

E.faecalis-E349 PLLVIEVNGLSSGQAGMILLPGGVAVAILSPFVGRLSDRFGDKRLIITGMTLMGLSTLFL 360

E.faecalis-SJ82 PLLVVEVNGLSSGQAGMILLPGGVAVAILSPFVGRLSDRFGDKRLIITGMTLMGLSTLFL 360

****:*******************************************************

E.faecalis-E349 STYASGASPLLVSVGVLGVGIAFAFTNSPANNAAVSALDADKVGVGMGIFQGALYLGAGT 420

E.faecalis-SJ82 STYASGASPLLVSVGVLGVGIAFAFTNSPANNAAVSALDADKVGVGMGIFQGALYLGAGT 420

************************************************************

E.faecalis-E349 GAGMIGALLSARRDATEPINPLYILDAMSYSDAFLAATGAILIALIAGLGLKKRG 475

E.faecalis-SJ82 GAGMIGALLSARRDATEPINPLYILDAMSYSDAFLAATGAILIALIAGLGLKKRG 475

*******************************************************

**(B)**

E.faecalis-E349 MKKDSKSKEMIQSEKRGSTRLLMMVLSLSVLVGSITADSVNPVLPLISKALEASKSQVSW 60

E.hirae-fas4 MKKDSKSNEMIQSEKRGSTRLLMMVLSLSVLVGAITADLVNPVLPLISKDLEASKSQVSW 60

E.faecalis-ZJ11066 MKKDSKSKEMIQSEKRGSTRLLMMVLSLSVLVGSITADSVNPVLPLISKALEASKSQVSW 60

E.avium-C674 MKKDSKSKEMIQSEKRGSTRLLMMVLSLSVLVGSITADSVNPVLPLISKDLEASKSQVSW 60

E.faecalis-838523 MKKDSKSKEMIQSEKRGSTRLLMMVLSLSVLVGXITADXVNPVLPLISKXLEASKSQVSW 60

S.aureus-W333 MKKDSKSKEMIQSEKRGSTRLLMMVLSLSVLVAAITVDLVNPVLPLISKDLEASKSQVSW 60

S.aureus-PTDrAP2 MKKDSKSKEMIQSEKRGSTRLLMMVLSLSVLVGAITSDLVNPVLPLISKDLEASKSQVSW 60

E.faecalis-973450 MKKDSKSKEMIQSEKRGSTRLLMMVLSLSVLVGAITXDJVNPVLPLISKDLEASKSQVSW 60

E.faecalis-981649 MKKDSKSKEMIQSEKRGSTRLLMMVLSLSVLVGAITSDIVNPVLPLISKDLEASKSQVSW 60

E.faecalis-SJ82 MKKDSKSKEMIQSEKRGSTRLLMMVLSLSVLVGAITSDIVNPVLPLISKDLEASKSQVSW 60

S.sciuri-G07 MKKDSKSKEMIQSEKRGSTRLLMMVLSLSVLVGAITSDIVNPVLPLISKDLEASKSQVSW 60

*******:************************. ** * ********** **********

E.faecalis-E349 IVSGIALVLAIGVPIYGRISDFFELRKLYIFAIMILASGSLLCAIAPNLPLLVLGRMVQG 120

E.hirae-fas4 IVSGIALVLAIGVPIYGRISDFFELRKLYIFAIMILASGSLLCAIATNLPLLVLGRMVQG 120

E.faecalis-ZJ11066 IVSGIALVLAIGVPIYGRISDFFELRKLYIFAIMILASGSLLCAIAPNLPLLVLGRMVQG 120

E.avium-C674 IVSGIALVLAIGVPIYGRISDFFELRKLYIFAIMILASGSLLCAIAPNLPLLVLGRMVQG 120

E.faecalis-838523 IVSGIALVLAIGVPIYGRISDFFELRKLYIFAIMILASGSLLCAIAPNLPLLVLGRMVQG 120

S.aureus-W333 IVSGIALVLAIGVPIYGRISDFFELRKLYIFAIMILASGSLLCAIAPNLPLLVLGRMVQG 120

S.aureus-PTDrAP2 IVSGIALVLAIGVPIYGRISDFFELRKLYIFAIMILASGSLLCAIATNLPLLVLGRMVQG 120

E.faecalis-973450 IVSGIALVLAIGVPIYGRISDFFELRKLYIFAIMILASGSLLCAIAPNLPLLVLGRMVQG 120

E.faecalis-981649 IVSGIALVLAIGVPIYGRISDFFELRKLYIFAIMILASGSLLCAIAPNLPLLVLGRMVQG 120

E.faecalis-SJ82 IVSGIALVLAIGVPIYGRISDFFELRKLYIFAIMILASGSLLCAIAPNLPLLVLGRMVQG 120

S.sciuri-G07 IVSGIALVLAIGVPIYGRISDFFELRKLYIFTIMILASGSLLCAIAPNLPLLVLGRMVQG 120

*******************************:************** *************

E.faecalis-E349 AGMSAIPVLSVIAISKVFPQGKRGGALGIIAGSIGVGTAAGPIFGGVVGQYLGWNALFWF 180

E.hirae-fas4 AGMSAIPVLSVIAISKVFPQEKRGGALGIIAGSIGVGTAAGPIFGGVVGQYLGWNALFWF 180

E.faecalis-ZJ11066 AGMSAIPVLSVIAISKVFPQGKRGGALGIIAGSIGVGTAAGPIFGGVVGQYLGWNALFWF 180

E.avium-C674 AGMSAIPVLSVIAISKVFPQGKRGGALGIIAGSIGVGTAAGPIFGGVVGQYLGWNALFWF 180

E.faecalis-838523 AGMSAIPVLSXIAISKVFPQGKRGGALGIIAGSIGVGTAAGPIFGGVVGQYLGWNALFWF 180

S.aureus-W333 AGMSAIPVLSVIAISKVFPQGKRGGALGIIAGSIGVGTAAGPIFGGVVGQYLGWNALFWF 180

S.aureus-PTDrAP2 AGMSAIPVLSVIAISKVFPQGKRGGALGIIAGSIGVGTAAGPIFGGVVGQYLGWNALFWF 180

E.faecalis-973450 AGMSAIPVLSXIAISKVFPQGKRGGALGIIAGSIGVGTAAGPIFGGVVGQYLGWNALFWF 180

E.faecalis-981649 AGMSAIPVLSVIAISKVFPQGKRGGALGIIAGSIGVGTAAGPIFGGVVGQYLGWNALFWF 180

E.faecalis-SJ82 AGMSAIPVLSVIAISKVFPQGKRGGALGIIAGSIGVGTAAGPIFGGVVGQYLGWNALFWF 180

S.sciuri-G07 AGMSAIPVLSVIAISKVFPQGKRGGALGIIAGSIGVGTAAGPIFGGVVGQYLGWNALFWF 180

********** ********* ***************************************

E.faecalis-E349 TFLLAIMIVIGAYYALPTIKPAESVGSNKNFDFIGGLFLGLTVGLLLFGITQGETSGFSS 240

E.hirae-fas4 TFLLAIMIVIGAYYALPTIKSAESVGSNKNFDFIGGLLLGLTVGLLLFGITQGETSGFSS 240

E.faecalis-ZJ11066 TFLLAIMIVIGAYYALPTIKPAESVGSNKNFDFIGGLFLGLTVGLLLFGITQGETSGFSS 240

E.avium-C674 TFLLAIMIVIGAYYALPTIKPAESVGSNKNFDFIGGLFLGLTVGLLLFGITQGETSGFSS 240

E.faecalis-838523 TFLLAIMIVIGAYYALPTIKPAESVGSNKNFDFIGGLFLGLTVGLLLFGITQGETSGFSS 240

S.aureus-W333 TFLLAIMIVIGAYYALPTIKPAESVGSNKNFDFIGGLFLGLTVGLLLFGITQGETSGFSS 240

S.aureus-PTDrAP2 TFLLAIMIVIGAYYALPTIKPAESVGSNKNFDFIGGLLLGLTVGLLLFGITQGETSGFSS 240

E.faecalis-973450 TFLLAIMIVIGAYYALPTIKPAESVGSNKNFDFIGGLFLGLTVGLLLFGITQGETSGFSS 240

E.faecalis-981649 TFLLAIMIVIGAYYALPTIKPAESVGSNKNFDFIGGLFLGLTVGLLLFGITQGETSGFSS 240

E.faecalis-SJ82 TFLLAIMIVIGAYYALPTIKPAESVGSNKNFDFIGGLFLGLTVGLLLFGITQGETSGFSS 240

S.sciuri-G07 TFLLAIMIVIGAYYALPTIKPAESVGSNKNFDFIGGLFLGLTVGLLLFGITQGETSGFSS 240

******************** ****************:**********************

E.faecalis-E349 FSSLTSLIGSVVALVGFIWRIVTAENPFVPPVLFNNKDYVNTVIIAFFSMFAYFAVLVFV 300

E.hirae-fas4 FSSLTSLIGSVVALVGFIWRIVTAENPFVPPVLFNNKDYVNTVIIAFFSMFAYFAVLVFV 300

E.faecalis-ZJ11066 FSSLTSLIGSVVALVGFIWRIVTAENPFVPPVLFNNKDYVNTVIIAFFSMFAYFAVLVFA 300

E.avium-C674 FSSLTSLIGSVVALVGFIWRIVTAENPFVPPVLFNNKDYVNTVIIAFFSMFAYFAVLVFV 300

E.faecalis-838523 FSSLTSLIGSVVALVGFIWRIVTAENPFVPPVLFNNKDYVNTVIIAFFSMFAYFAVLVFV 300

S.aureus-W333 FSSLTSLIGSVVALVGFIWRIVTAENPFVPPVLFNNKDYVNTVIIAFFSMFAYFAVLVFV 300

S.aureus-PTDrAP2 FSSLTSLIGSVVALVGFIWRIVTAENPFVPPVLFNNKDYVNTVIIAFFSMFAYFAVLVFV 300

E.faecalis-973450 FSSLTSLIGSVVALVGFIWRIVTAENPFVPPVLFNNKDYVNTVIIAFFSMFAYFAVLVFV 300

E.faecalis-981649 FSSLTSLIGSVVALVGFIWRIVTAENPFVPPVLFNNKDYVNTVIIAFFSMFAYFAVLVFV 300

E.faecalis-SJ82 FSSLTSLIGSVVALVGFIWRIVTAENPFVPPVLFNNKDYVNTVIIAFFSMFAYFAVLVFV 300

S.sciuri-G07 FSSLTSLIGSVVALVGFIWRIVTAENPFVPPVLFNNKDYVNTVIIAFFSMFAYFAVLVFV 300

***********************************************************.

E.faecalis-E349 PLLVIEVNGLSSGQAGMILLPGGVAVAILSPFVGRLSDRFGDKRLIITGMTLMGLSTLFL 360

E.hirae-fas4 PLLVIEVNGLSSGQAGMILLPGGVAVAILSPFVGRLSDRFGDKRLIITGMTLMGLSTLFL 360

E.faecalis-ZJ11066 PLLVIEVNGLSSGQAGMILLPGGVAVAILSPFVGRLSDRFGDKRLIITGMTLMGLSTLFL 360

E.avium-C674 PLLVIEVNGLSSGQAGMILLPGGVAVAILSPFVGRLSDRFGDKRLIITGMTLMGLSTLFL 360

E.faecalis-838523 PLLVXEVNGLSSGQAGMILLPGGVAVAILSPFVGRLSDRFGDKRLIITGMTLMGLSTLFL 360

S.aureus-W333 PLLVVEVNGLSSGQAGMILLTGGVAVAILSPFVGRLSDRFGDKRLIITGMTLMGLSTLFL 360

S.aureus-PTDrAP2 PLLVVEVNGLSSGQAGMILLPGGVAVAILSPFVGRLSDRFGDKRLIITGMTLMGLSTLFL 360

E.faecalis-973450 PLLVVEVNGLSSGQAGMILLPGGVAVAILSPFVGRLSDRFGDKRLIITGMTLMGLSTLFL 360

E.faecalis-981649 PLLVVEVNGLSSGQAGMILLPGGVAVAILSPFVGRLSDRFGDKRLIITGMTLMGLSTLFL 360

E.faecalis-SJ82 PLLVVEVNGLSSGQAGMILLPGGVAVAILSPFVGRLSDRFGDKRLIITGMTLMGLSTLFL 360

S.sciuri-G07 PLLVVEVNGLSSGQAGMILLPGGVAVAILSPFVGRLSDRFGDKRLIITGMTLMGLSTLFL 360

**** *************** ***************************************

E.faecalis-E349 STYASGASPLLVSVGVLGVGIAFAFTNSPANNAAVSALDADKVGVGMGIFQGALYLGAGT 420

E.hirae-fas4 STYASGASPLLVSVGVLGVGIAFAFTNSPANNAAVSALEADKVGVGMGIFQGALYLGAGT 420

E.faecalis-ZJ11066 STYASGASPLLVSVGVLGVGIAFAFTNSPANNAAVSALDADKVGVGMGIFQGALYLGAGT 420

E.avium-C674 STYASGASPLLVSVGVLGVGIAFAFTNSPANNAAVSALDADKVGVGMGIFQGALYLGAGT 420

E.faecalis-838523 STYASGASPLLVSVGVLGVGIAFAFTNSPANNAAVSALDADKVGVGMGIFQGALYLGAGT 420

S.aureus-W333 STYASGASPLLVSVGVLGVGIAFAFTNSPANNAAVSALDADKVGVGMGIFQGALYLGAGT 420

S.aureus-PTDrAP2 STYASGASPLLVSVGVLGVGIAFAFTNSPANNAAVSALDADKVGVGMGIFQGALYLGAGT 420

E.faecalis-973450 STYASGASPLLVSVGVLGVGIAFAFTNSPANNAAVSALDADKVGVGMGIFQGALYLGAGT 420

E.faecalis-981649 STYASGASPLLVSVGVLGVGIAFAFTNSPANNAAVSALDADKVGVGMGIFQGALYLGAGT 420

E.faecalis-SJ82 STYASGASPLLVSVGVLGVGIAFAFTNSPANNAAVSALDADKVGVGMGIFQGALYLGAGT 420

S.sciuri-G07 STYASGASPLLVSVGVLGVGIAFAFTNSPANNAAVSALDADKVGVGMGIFQGALYLGAGT 420

**************************************:*********************

E.faecalis-E349 GAGMIGALLSARRDATEPINPLYILDAMSYSDAFLAATGAILIALIAGLGLKKRG 475

E.hirae-fas4 GAGMIGALLSARRDATEPINPLYILDAMSYSDAFLAATGAILIALIAGLGLKKRG 475

E.faecalis-ZJ11066 GAGMIGALLSARRDATEPINPLYILDAMSYSDAFLAATGAILIALIAGLGLKKRG 475

E.avium-C674 GAGMIGALLSARRDATEPINPLYILDAMSYSDAFLAATGAILIALIAGLGLKKRG 475

E.faecalis-838523 GAGMIGALLSARRDATEPINPLYILDAMSYSDAFLAATGAILIALIAGLGLKKRG 475

S.aureus-W333 GAGMIGALLSARRDATEPINPLYILDAMSYSDAFLAATGAILIALIAGLGLKKRG 475

S.aureus-PTDrAP2 GAGMIGALLSARRDATEPINPLYILDAMSYSDAFLAATGAILIALIAGLGLKKRG 475

E.faecalis-973450 GAGMIGALLSARRDATEPINPLYILDAMSYSDAFLAATGAILIALIAGLGLKKRG 475

E.faecalis-981649 GAGMIGALLSARRDATEPINPLYILDAMSYSDAFLAATGAILIALIAGLGLKKRG 475

E.faecalis-SJ82 GAGMIGALLSARRDATEPINPLYILDAMSYSDAFLAATGAILIALIAGLGLKKRG 475

S.sciuri-G07 GAGMIGALLSARRDATEPINPLYILDAMSYSDAFLAATGAILIALIAGLGLKKRG 475

*******************************************************

**Fig. S2** Amino acid sequence alignment of chloramphenicol/florfenicol efflux MFS transporter FexA of *E. faecalis* strains E349 and SJ82 (a), and among ten FexA variants (b). Strain SJ82 analyzed in this study has identical sequence to strain 981649. Amino acids different from prototype strain E349 are shown in yellow. Asterisk indicates identical amino acid.
