# Supplementary material for: Drug Resistance Determinants in Clinical Isolates of Enterococcus faecalis in Bangladesh: Identification of Oxazolidinone Resistance Gene optrA in ST59 and ST902 Lineages
Source: Microorganisms. 2020 Aug 14;8(8):1240. doi: 10.3390/microorganisms8081240 (PMC7463919; doi:10.3390/microorganisms8081240)
Supplement: Supplementary file 1 [file microorganisms-08-01240-s001.zip › Supplementary Material SRoy/FigS3-RNaseJ SRoy.docx]

**(a)**

981649 ATGAAAGTAAACATAAAAAATAACGAAACTGGCGTTTTTGCAATCGGTGGTTTAGGCGAA 60

SJ82 ATGAAAGTAAACATAAAAAATAACGAAACTGGCGTTTTTGCAATCGGTGGTTTAGGCGAA 60

743142 ATGAAAGTAAACATAAAAAATAACGAAACTGGCGTTTTTGCAATCGGTGGTTTAGGCGAA 60

************************************************************

981649 ATTGGAAAAAACACATATGGGGTACAATTCCAAGATGAAATTATCATTATTGATGCTGGG 120

SJ82 ATTGGAAAAAACACATATGGGGTACAATTCCAAGATGAAATTATCATTATTGATGCTGGG 120

743142 ATTGGAAAAAACACATATGGGGTACAATTCCAAGATGAAATTATCATTATTGATGCTGGG 120

************************************************************

981649 ATCAAATTCCCAGAAGATGATTTACTTGGGATTGACTACGTGATTCCAGATTATAGCTAC 180

SJ82 ATCAAATTCCCAGAAGATGATTTACTTGGGATTGACTACGTGATTCCAGATTATAGCTAC 180

743142 ATCAAATTCCCAGAAGATGATTTACTTGGGATTGACTACGTGATTCCAGATTATAGCTAC 180

************************************************************

981649 ATCGTTCAAAATTTGCATAAAGTCAAAGCACTCGTTATTACCCACGGTCATGAAGACCAT 240

SJ82 ATCGTTCAAAATTTGCATAAAGTCAAAGCACTCGTTATTACCCACGGTCATGAAGACCAT 240

743142 ATCGTTCAAAATTTGCATAAAGTCAAAGCACTCGTTATTACCCACGGTCATGAAGACCAT 240

************************************************************

981649 ATTGGTGGCGTTCCTTATCTTTTACGTCAAGCGAATATCCCAATCTATGCGGGACCACTA 300

SJ82 ATTGGTGGCGTTCCTTATCTTTTACGTCAAGCGAATATCCCAATCTATGCGGGACCACTA 300

743142 ATTGGTGGCGTTCCTTATCTTTTACGTCAAGCGAATATCCCAATCTATGCGGGACCACTA 300

************************************************************

981649 GCCTTGGCTTTAATTACCAACAAACTAGATGAACATGGTTTATTGCGAGAAGCAGAATTA 360

SJ82 GCCTTGGCTTTAATTACCAACAAACTAGATGAACATGGTTTATTGCGAGAAGCGGAATTA 360

743142 GCCTTGGCTTTAATTACCAACAAACTAGATGAACATGGTTTATTGCGAGAAGCGGAATTA 360

***************************************************** ******

981649 CACGAAATTAATGAAGATACGGTGATTCGTTTTAGAAAAACAGCGATTAGCTTTTTCCGG 420

SJ82 CACGAAATTAATGAAGATACGGTGATTCGTTTTAGAAAAACAGCGATTAGTTTTTTCCGG 420

743142 CACGAAATTAATGAAGATACGGTGATTCGTTTTAGAAAAACAGCGATTAGCTTTTTCCGG 420

************************************************** *********

981649 ACGACTCACAGTATTCCTGATGCCTTGGGCGTTGTGGTTAAAACGCCTTCTGGTAACATT 480

SJ82 ACGACTCACAGTATTCCTGATGCCTTGGGCGTTGTGGTTAAAACGCCTTCTGGTAACATT 480

743142 ACGACTCACAGTATTCCTGATGCCTTGGGCGTTGTGGTTAAAACGCCTTCTGGTAACATT 480

************************************************************

981649 GTCGCAACAGGTGACTTTAAGTTTGACTTTACACCCGTTGGCGAGCCAGCCAACTTACAC 540

SJ82 GTCGCAACAGGTGACTTTAAGTTTGACTTTACACCCGTTGGTGAGCCAGCCAACTTACAC 540

743142 GTCGCAACAGGTGACTTTAAGTTTGACTTTACACCCGTTGGTGAGCCAGCCAACTTACAC 540

***************************************** ******************

981649 AGAATGGCGAAATTAGGTGAAGAAGGCGTCCTTTGTCTTCTTTCAGACAGTACCAATGCT 600

SJ82 AGAATGGCGAAATTAGGTGAAGAAGGCGTCCTTTGTCTTCTTTCAGACAGTACCAATGCT 600

743142 AGAATGGCGAAATTAGGTGAAGAAGGCGTCCTTTGTCTTCTTTCAGACAGTACCAATGCT 600

************************************************************

981649 GAAATACCCACCTTTACAAAATCCGAAAAAACAATTGGGACCTCTATTTTAAAAATCTTT 660

SJ82 GAAATACCCACCTTTACAAAATCCGAAAAAACAATTGGGACCTCTATTTTAAAAATCTTT 660

743142 GAAATACCCACCTTTACAAAATCCGAAAAAACAATCGGGACCTCTATTTTAAAAATCTTT 660

*********************************** ************************

981649 GAAAAAATTGATGGTCGGATTATCTTTGCCAGTTTTGCTTCAAACATTTTCCGTTTACAA 720

SJ82 GAAAAAATTGATGGTCGGATTATCTTTGCCAGTTTTGCTTCAAACATTTTCCGTTTACAA 720

743142 GAAAAAATTGATGGTCGGATTATCTTTGCCAGTTTCGCTTCAAACATTTTCCGTTTACAA 720

*********************************** ************************

981649 CAAGCTGCTGATGCAGCCGTGAAAACCGGTCGCAAAATTGCTGTTTTTGGTCGTAGCATG 780

SJ82 CAAGCTGCTGATGCAGCCGTGAAAACAGGTCGCAAAATTGCTGTTTTTGGTCGTAGCATG 780

743142 CAAGCTGCTGATGCAGCTGTGAAAACCGGTCGCAAAATTGCTGTTTTTGGTCGTAGCATG 780

***************** ******** *********************************

981649 GAAAATGCCATTGTCAATGGCGAACGCTTAGGCTACATCAAAGTGCCAAAAGGAACGTTT 840

SJ82 GAAAATGCCATTGTCAATGGCGAACGCTTAGGCTACATCAAAGTGCCAAAAGGAACGTTT 840

743142 GAAAATGCCATTGTCAATGGCGAACGCTTAGGCTACATCAAAGTGCCAAAAGGAACGTTT 840

************************************************************

981649 GTCGATGCGGCGGAACTGAATCAATTGCCAGCAAATGAAACAATGATTTTATGTACTGGC 900

SJ82 GTCGATGCGGCGGAACTGAATCAATTGCCAGCAAATGAAACAATGATTTTATGTACTGGC 900

743142 GTCGATGCGGCGGAACTGAATCAATTGCCAGCAAATGAAACAATGATTTTATGTACTGGC 900

************************************************************

981649 TCTCAAGGAGAACCTATGGCCGCGCTTAGTCGGATTGCTAACGGTACACATCGCCAAATT 960

SJ82 TCTCAAGGAGAACCTATGGCCGCGCTTAGTCGGATTGCTAACGGTACACATCGCCAAATT 960

743142 TCTCAAGGAGAACCTATGGCCGCGCTTAGTCGGATTGCTAACGGTACACACCGTCAAATT 960

************************************************** ** ******

981649 AGTATTCAACCAGGCGATACAGTTGTTTTCTCAAGTTCACCAATTCCTGGGAATACGACA 1020

SJ82 AGTATTCAACCAGGCGATACGGTTGTTTTCTCAAGTTCACCAATTCCTGGGAATACGACA 1020

743142 AGTATTCAACCAGGCGATACGGTTGTTTTCTCAAGCTCACCAATTCCTGGGAATACGACA 1020

******************** ************** ************************

981649 AGCGTTAACCGTTTAATCAACTTATTATCTGAAGCCGGTGCAGAAGTTATCCACGGAAAA 1080

SJ82 AGCGTTAACCGTTTAATCAACTTATTATCTGAAGCCGGTGCAGAAGTTATCCACGGAAAA 1080

743142 AGTGTTAACCGTTTAATCAACTTATTATCTGAAGCCGGTGCGGAAGTTATCCACGGAAAA 1080

** ************************************** ******************

981649 ATCAATAATATCCATACTTCTGGTCACGGTGGACAAGAAGAGCAAAAATTAATGCTTCGC 1140

SJ82 ATCAATAATATCCATACTTCTGGTCACGGTGGACAAGAAGAGCAAAAATTAATGCTTCGT 1140

743142 ATCAATAATATCCATACTTCTGGTCACGGTGGACAAGAAGAGCAAAAATTAATGCTTCGC 1140

***********************************************************

981649 TTGATGAAGCCTAAATATTTCATGCCTGTTCATGGTGAATTTAGAATGTTAAAAATCCAT 1200

SJ82 TTGATGAAGCCTAAATATTTCATGCCTGTTCATGGTGAATTTAGAATGTTAAAAATCCAT 1200

743142 TTGATGAAGCCTAAATATTTCATGCCTGTTCATGGTGAATTTAGAATGTTAAAAATCCAT 1200

************************************************************

981649 GCTTCGCTCGCTCAAGATACAGGGGTTCCTGAAGAAAATTGCTTTATCATGGGCAACGGC 1260

SJ82 GCTTCGCTCGCTCAAGATACAGGGGTTCCTGAAGAAAATTGCTTTATCATGGGCAACGGC 1260

743142 GCTTCGCTCGCTCAAGATACAGGGGTTCCTGAAGAAAATTGCTTTATCATGGGCAACGGC 1260

************************************************************

981649 GATGTCTTAGCTTTAACGGCTGATAGCGCACGTCCTGCTGGTCACTTCAACGCTAACGAC 1320

SJ82 GATGTCTTAGCTTTAACGGCTGATAGCGCACGTCCTGCTGGTCACTTCAACGCTAACGAC 1320

743142 GATGTCTTAGCTTTAACGGCTGATAGCGCACGTCCTGCTGGTCACTTCAACGCTAATGAC 1320

******************************************************** ***

981649 GTGTACGTTGATGGAAATGGTGTCGGCGATATCGGGAATGTTGTTTTACGGGATCGTCGT 1380

SJ82 GTGTACGTTGATGGAAATGGTGTCGGTGATATCGGGAATGTTGTTTTACGGGATCGTCGT 1380

743142 GTGTACGTTGATGGAAATGGTGTCGGCGATATCGGGAATGTTGTTTTACGGGATCGTCGT 1380

************************** *********************************

981649 ATCTTATCTGAAGAAGGTTTAGTTTTAGCGGTAGCAACGGTTGATATTAAAAATAAAGAA 1440

SJ82 ATCTTATCTGAAGAAGGTTTAGTTTTAGCGGTAGCAACGGTTGATATTAAAAATAAAGAA 1440

743142 ATCTTATCTGAAGAAGGTTTAGTTTTAGCGGTAGCAACAGTTGATATTAAAAATAAAGAA 1440

************************************** *********************

981649 ATTATGGCTGGTCCAGACATTCTTTCCCGTGGTTTTGTCTACATGCGTGAGTCTGGTGAT 1500

SJ82 ATTATGGCTGGTCCAGACATTCTTTCCCGTGGTTTTGTCTACATGCGTGAGTCTGGTGAT 1500

743142 ATTATGGCTGGTCCAGACATTCTTTCCCGTGGTTTTGTCTACATGCGTGAGTCTGGTGAT 1500

************************************************************

981649 ATGATTCATGAAGGCCAACGTCTTTTATTCAATGCTCTACGTGAAGCAATGAAAGATAAA 1560

SJ82 ATGATTCATGAAGGCCAACGTCTTTTATTCAATGCTCTACGTGAAGCAATGAAAGATAAA 1560

743142 ATGATTCATGAAGGCCAACGTCTTTTATTCAATGCTCTACGTGAAGCAATGAAAGATAAA 1560

************************************************************

981649 AACTGTACCGAAGCAAAATTAGGCGAAGCCATGACGACCGCTTTACAGCCTTTCTTGTTT 1620

SJ82 AACTGTACCGAAGCAAAATTAGGCGAAGCCATGACGACCGCTTTACAGCCTTTCTTGTTT 1620

743142 AACTGTACCGAAGCAAAATTAGGCGAAGCCATGACGACCGCTTTACAGCCTTTCTTGTTT 1620

************************************************************

981649 GAACAAACAGAACGTCATCCAATGATTCTGCCAATGATTCTGCCAATGATTATGACTGCC 1680

SJ82 GAACAAACAGAACGTCATCCAATGATTCTGCCAATGATTATGACTGCC------------ 1668

743142 GAACAAACAGAACGTCATCCAATGATTCTGCCAATGATTATGACTGCC------------ 1668

*************************************** ** *

981649 ACAGTTAGTGATCAATAA 1698

SJ82 ACAGTTAGTGATCAATAA 1686

743142 ACAGTTAGTGATCAATAA 1686

******************

**(b)**

SJ82 MKVNIKNNETGVFAIGGLGEIGKNTYGVQFQDEIIIIDAGIKFPEDDLLGIDYVIPDYSY 60

743142 MKVNIKNNETGVFAIGGLGEIGKNTYGVQFQDEIIIIDAGIKFPEDDLLGIDYVIPDYSY 60

981649 MKVNIKNNETGVFAIGGLGEIGKNTYGVQFQDEIIIIDAGIKFPEDDLLGIDYVIPDYSY 60

************************************************************

SJ82 IVQNLHKVKALVITHGHEDHIGGVPYLLRQANIPIYAGPLALALITNKLDEHGLLREAEL 120

743142 IVQNLHKVKALVITHGHEDHIGGVPYLLRQANIPIYAGPLALALITNKLDEHGLLREAEL 120

981649 IVQNLHKVKALVITHGHEDHIGGVPYLLRQANIPIYAGPLALALITNKLDEHGLLREAEL 120

************************************************************

SJ82 HEINEDTVIRFRKTAISFFRTTHSIPDALGVVVKTPSGNIVATGDFKFDFTPVGEPANLH 180

743142 HEINEDTVIRFRKTAISFFRTTHSIPDALGVVVKTPSGNIVATGDFKFDFTPVGEPANLH 180

981649 HEINEDTVIRFRKTAISFFRTTHSIPDALGVVVKTPSGNIVATGDFKFDFTPVGEPANLH 180

************************************************************

SJ82 RMAKLGEEGVLCLLSDSTNAEIPTFTKSEKTIGTSILKIFEKIDGRIIFASFASNIFRLQ 240

743142 RMAKLGEEGVLCLLSDSTNAEIPTFTKSEKTIGTSILKIFEKIDGRIIFASFASNIFRLQ 240

981649 RMAKLGEEGVLCLLSDSTNAEIPTFTKSEKTIGTSILKIFEKIDGRIIFASFASNIFRLQ 240

************************************************************

SJ82 QAADAAVKTGRKIAVFGRSMENAIVNGERLGYIKVPKGTFVDAAELNQLPANETMILCTG 300

743142 QAADAAVKTGRKIAVFGRSMENAIVNGERLGYIKVPKGTFVDAAELNQLPANETMILCTG 300

981649 QAADAAVKTGRKIAVFGRSMENAIVNGERLGYIKVPKGTFVDAAELNQLPANETMILCTG 300

************************************************************

SJ82 SQGEPMAALSRIANGTHRQISIQPGDTVVFSSSPIPGNTTSVNRLINLLSEAGAEVIHGK 360

743142 SQGEPMAALSRIANGTHRQISIQPGDTVVFSSSPIPGNTTSVNRLINLLSEAGAEVIHGK 360

981649 SQGEPMAALSRIANGTHRQISIQPGDTVVFSSSPIPGNTTSVNRLINLLSEAGAEVIHGK 360

************************************************************

SJ82 INNIHTSGHGGQEEQKLMLRLMKPKYFMPVHGEFRMLKIHASLAQDTGVPEENCFIMGNG 420

743142 INNIHTSGHGGQEEQKLMLRLMKPKYFMPVHGEFRMLKIHASLAQDTGVPEENCFIMGNG 420

981649 INNIHTSGHGGQEEQKLMLRLMKPKYFMPVHGEFRMLKIHASLAQDTGVPEENCFIMGNG 420

************************************************************

SJ82 DVLALTADSARPAGHFNANDVYVDGNGVGDIGNVVLRDRRILSEEGLVLAVATVDIKNKE 480

743142 DVLALTADSARPAGHFNANDVYVDGNGVGDIGNVVLRDRRILSEEGLVLAVATVDIKNKE 480

981649 DVLALTADSARPAGHFNANDVYVDGNGVGDIGNVVLRDRRILSEEGLVLAVATVDIKNKE 480

************************************************************

SJ82 IMAGPDILSRGFVYMRESGDMIHEGQRLLFNALREAMKDKNCTEAKLGEAMTTALQPFLF 540

743142 IMAGPDILSRGFVYMRESGDMIHEGQRLLFNALREAMKDKNCTEAKLGEAMTTALQPFLF 540

981649 IMAGPDILSRGFVYMRESGDMIHEGQRLLFNALREAMKDKNCTEAKLGEAMTTALQPFLF 540

************************************************************

SJ82 EQTERHPMILPMI----MTATVSDQ 561

743142 EQTERHPMILPMI----MTATVSDQ 561

981649 EQTERHPMILPMILPMIMTATVSDQ 565

************* ********

**Fig. S3** Alignment of nucleotide (a) and amino acid (b) sequences of RNase J family beta-CASP ribonuclease gene of *E. faecalis* strains SJ82, 981649 and 743142. Asterisk indicates identical nucleotide/amino acid, dash denotes gap. Nucleotides/amino acids of strain 981649 and 743142 that are different from those of SJ82 is indicated in yellow.
